# Management of Multiple Impacted Maxillary Teeth: A Case Report

**DOI:** 10.7759/cureus.82698

**Published:** 2025-04-21

**Authors:** Rahaf T Alosaimi, Mohammed A Barashi

**Affiliations:** 1 Periodontics and Implant Dentistry, King Faisal Hospital, Makkah, SAU; 2 Preventive Dentistry, Umm Al-Qura University, Makkah, SAU

**Keywords:** canine, dentistry, exposure, impaction, incisors

## Abstract

For many years, diagnosing and managing multiple impacted teeth has been a challenge for orthodontists. To effectively manage such complex cases, an interdisciplinary approach is required. The outcome will be favorable and aesthetically pleasing if the proper orthodontic treatment plan and surgical approach are chosen. An 11-year-old female patient presented to our orthodontic clinic with her parents. Upon clinical examination, the maxillary left central incisor, lateral incisor, and canine bilaterally had not erupted (teeth #13, 21, 22, 23). The case was managed by surgical exposure and orthodontic treatment. The treatment of impacted teeth is crucial for both esthetics and functionality, and it requires the expertise of several clinicians. The successful treatment of impacted teeth depends on precise localization, cautious soft tissue management, optimal surgical approach selection, and controlled orthodontic traction in direction and force.

## Introduction

The management of multiple tooth impactions has long posed an intriguing challenge for orthodontists, both in diagnosis and treatment [1]. Among the impacted teeth, mandibular third molars are the most frequent, followed by maxillary third molars, maxillary canines, maxillary and mandibular premolars, maxillary incisors, mandibular canines, mandibular incisors, maxillary and mandibular first molars, and maxillary and mandibular second molars [2-4].

Genetics are thought to play a significant role in tooth impaction, with several contributing factors, including differences in tooth size and arch length, early loss or prolonged retention of deciduous canines, abnormal tooth bud position, alveolar cleft presence, ankylosis, cysts, neoplasms, root dilaceration, iatrogenic causes, and idiopathic conditions [4].

A thorough diagnosis is necessary to identify and prevent impaction. This includes a full family history, clinical examination with palpation between the ages of 9 and 10 years, and comprehensive radiographic examination [5].

Maxillary canines typically develop near the nasal cavity, resulting in the longest eruption path among permanent teeth. Approximately 1.5% of the population may exhibit ectopic eruption paths of canines toward the palate [6]. This dental anomaly not only prevents spontaneous eruption but also increases the risk of root resorption in neighboring teeth [7]. Canine impaction prevalence in children aged 7-13 is around 2.2%, with females experiencing impactions at twice the rate of males [7,8].

An interdisciplinary approach is necessary for successful management. Selecting the right orthodontic treatment plan and surgical technique will produce a predictable, stable, and aesthetic outcome. Four criteria should be evaluated to determine the appropriate method for uncovering the tooth: the labiolingual position of the impacted crown, the vertical position of the tooth relative to the mucogingival junction, the amount of attached gingiva around the impacted tooth, and the mesiodistal position of the crown. Several surgical techniques have been described to expose these impacted teeth. Three methods can be employed: excisional uncovering or gingivectomy, an apically positioned flap, and the closed eruption technique [9].

We present a case with multiple impacted maxillary teeth of an 11-year-old female patient who had her primary teeth extracted and was treated with extraction and surgical exposure.

## Case presentation

An 11-year-old female patient presented to our private clinic in Makkah, Saudi Arabia, with her parents. Her chief complaint was, "My permanent front teeth have not erupted yet." Her general physical status was normal. Her facial profile was concave, with average facial height and competent lips.

**Figure 1:**
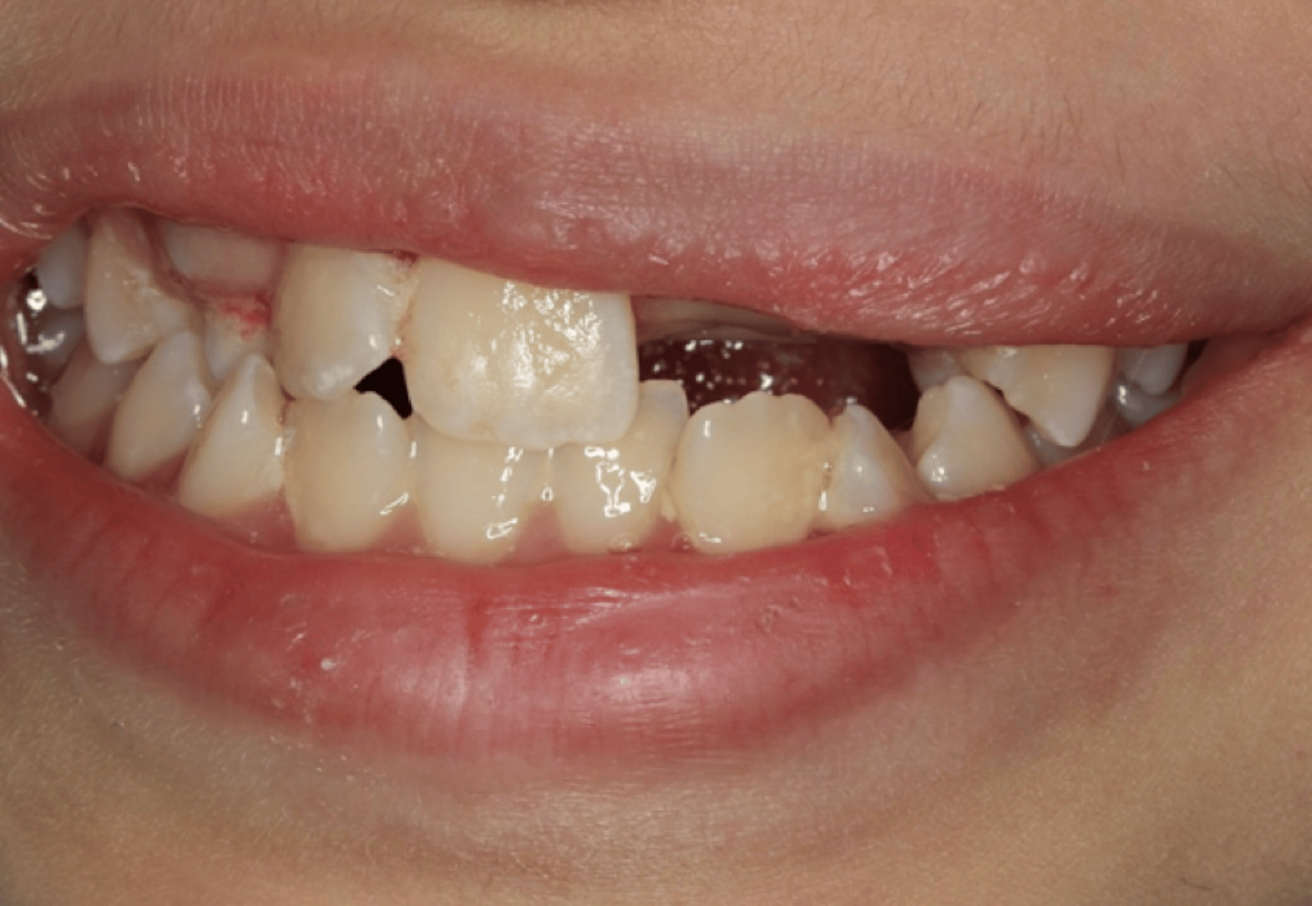
Preoperative smile

Her dental history revealed multiple restorations (teeth #26, 46), extractions of primary teeth (#54, 55, 65, 64, 75), and orthodontic treatment with a face-mask combined with a Hyrax expander to correct her skeletal Class III malocclusion two years ago. She brushes twice daily and flosses once daily. Extraoral examination revealed no pathology or abnormalities (Figure 1).

**Figure 2:**
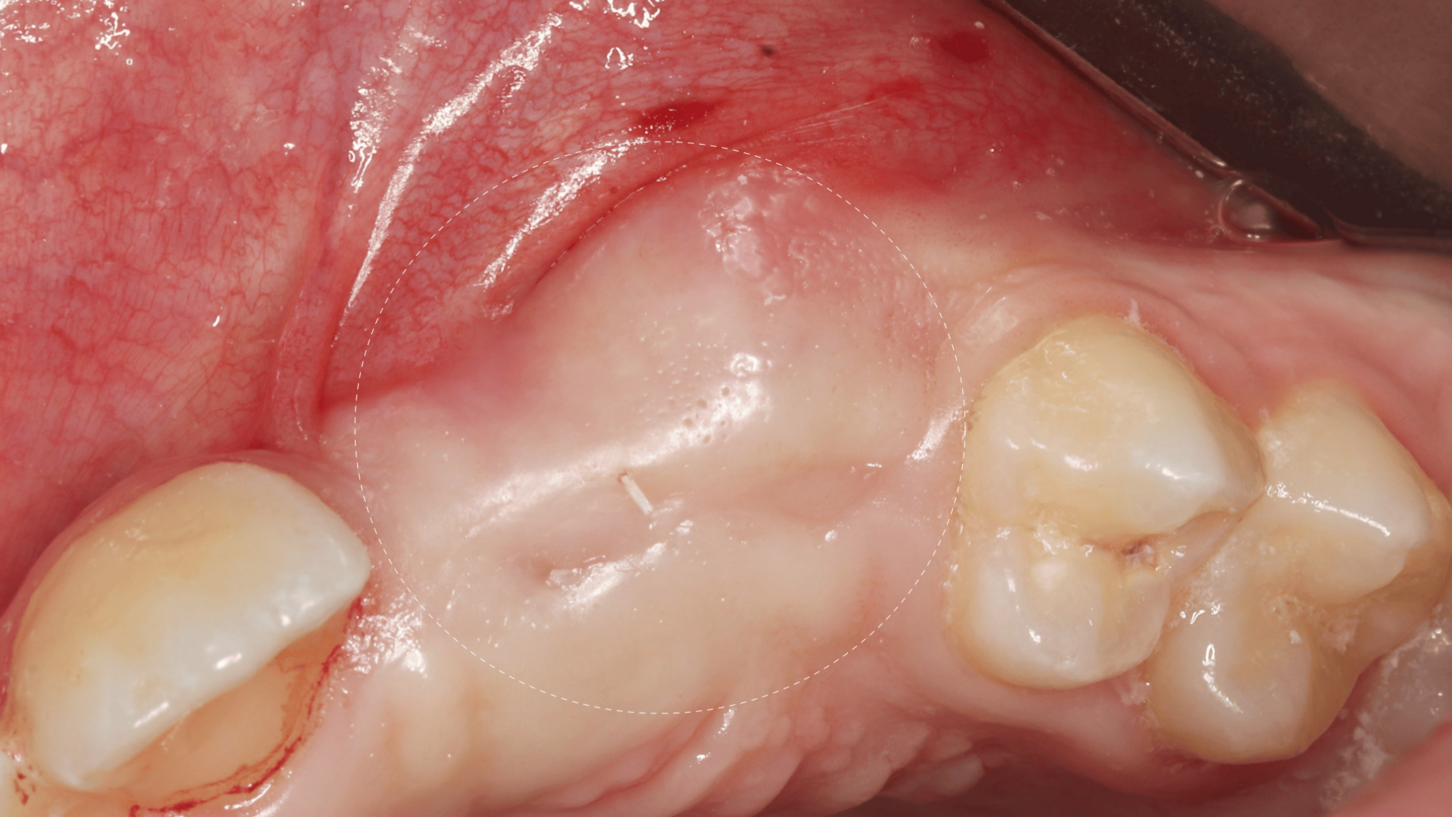
Preoperative intraoral clinical photograph with unerupted left central incisor, lateral incisor, and canine (teeth #21, 22, 23)

Upon clinical examination, the maxillary left central incisor, lateral incisor, and canines were missing (teeth #13, 21, 22, 23). The space available in the maxillary arch was around 13 mm. Anteroposteriorly, the molars and canines were in a Class I relationship, and overjet ranged from 0 to 2 mm. Vertically, the overbite was 3 mm. No crossbite was noted. Oral hygiene was fair. Intraoral examination revealed no pathology (Figure 2).

**Figure 3:**
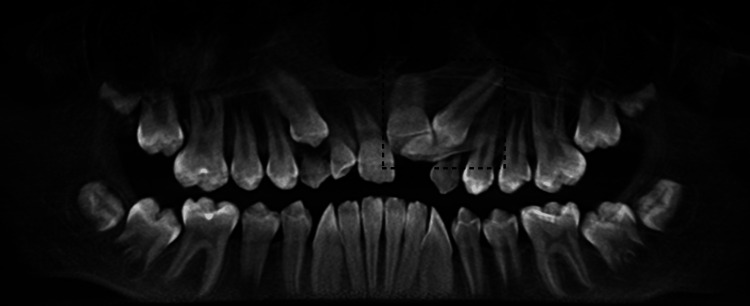
Retained maxillary primary right and left canines and an impaction of maxillary permanent left central incisor, lateral incisor, and both canines

**Figure 4:**
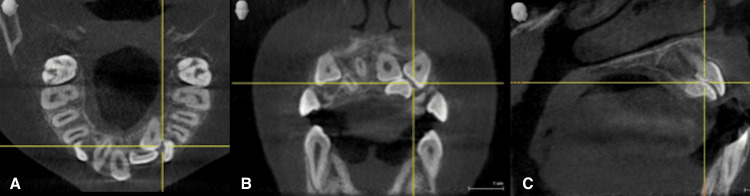
(A) Axial view, (B) coronal view, and (C) sagittal view, showing retained maxillary primary right and left canines and an impaction of maxillary permanent left central incisor, lateral incisor and both canines

Orthopantomogram confirmed the presence of retained maxillary primary canines and impacted maxillary left central incisor, lateral incisor, and canines (Figure 3). Therefore, further imaging was obtained with cone-beam computed tomography to localize the position of the impacted teeth (Figure 4). Cone-beam computed tomography revealed vertical impaction of the maxillary left central incisor, underlined by horizontal impaction of the maxillary left lateral incisor and vertical impaction of the maxillary left canine.

Treatment objectives comprised surgical exposure of the impacted maxillary left central incisor and canine, extraction of the maxillary left lateral incisor, and referral to the orthodontics department for forced eruption of the impacted maxillary left central incisor and canine, along with correction of deep bite, maintenance of molar relation, and achieving a pleasing profile and smile.

**Figure 5:**
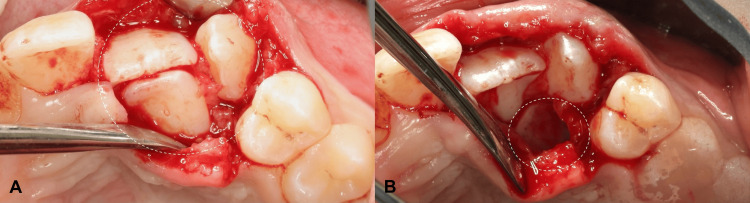
(A) Surgical exposure of impacted left central incisor, lateral incisor, and canine (teeth #21, 22, 23). (B) After surgical extraction of left lateral incisor (tooth #22)

After counseling the patient and parents regarding the treatment plan, informed consent was obtained. Surgical exposure of the impacted teeth was performed in the Department of Periodontics. The impacted teeth were positioned coronal to the mucogingival line, with sufficient keratinized mucosa and a thick phenotype. Topical and local anesthesia were applied to the buccal and palatal areas. Using a number 15C scalpel blade, a gingivectomy or excision technique was performed buccally, while a full-thickness palatal flap was raised using the same blade. Teeth #21 and #23 were exposed (Figure 5-A), while tooth #22 was extracted (Figure 5-B), and the flap was sutured back using the simple interrupted suture technique with 5-0 polyglycolic acid suture. Postoperative instructions were provided, and paracetamol 250 mL syrup every six hours for five days was prescribed. After one week, sutures were removed; healing was favorable, with no complications. The patient was referred to the orthodontic department for comprehensive orthodontic treatment, with continuing follow-up in the periodontal department every three months.

## Discussion

Depending on which tooth is involved, an unerupted or impacted tooth can cause significant functional and/or aesthetic problems. When treating impacted maxillary anterior teeth, especially canines, an interdisciplinary approach incorporating orthodontic, periodontal, restorative, and oral surgical procedures is typically necessary. Prudent treatment planning is essential to achieve the various treatment goals [10].

The outcome of the therapy is greatly dependent on the conservative surgical technique used and the orthodontic forces applied. The quality of the periodontium, an assessment of the height of the keratinized tissue band, and the position of the emergence point are the primary factors influencing the periodontal prognosis of the affected teeth [11].

To identify the precise location of the erupting position of the impacted tooth, the use of three-dimensional imaging with CBCT has shown superiority over traditional radiographs [12].

In the present case, the position of the central incisor and canine was coronal to the mucogingival line, canine predominance was detectable, the amount of keratinized mucosa was sufficient, and there was no bone obstruction. Thus, the gingivectomy technique was the most efficient and minimally invasive method to be chosen.

The optimal alignment of an impacted tooth depends on a number of factors, including the affected tooth's position and orientation, the degree of root completeness, and the amount of space available in the arch for the impacted tooth [13].

The horizontal position of the lateral incisor and limited arch space prompted the decision to extract the tooth to create space for the canine and central incisor.

Future mucogingival complications may be avoided with careful planning of mucogingival interceptive procedures performed at the appropriate time, with a suitable technique [14].

Careful case evaluation and the planning of suitable treatment biomechanics, supported by three-dimensional analyses, ensured successful treatment outcomes with a smile that is aesthetically pleasing and a harmonic occlusal relationship [15].

## Conclusions

The treatment of impacted teeth is crucial for both esthetics and functionality, and it requires the expertise of several clinicians. Multiple surgical and orthodontic procedures can be utilized to expose impacted maxillary teeth, depending on their location and angulation. The successful treatment of impacted teeth depends on precise localization, careful soft tissue management, optimal surgical approach selection, and appropriate orthodontic traction in direction and force.
